# Early Response of CD8+ T Cells in COVID-19 Patients

**DOI:** 10.3390/jpm11121291

**Published:** 2021-12-03

**Authors:** Deni Ramljak, Martina Vukoja, Marina Curlin, Katarina Vukojevic, Maja Barbaric, Una Glamoclija, Bejana Purisevic, Olivera Peric, Violeta Soljic

**Affiliations:** 1 Laboratory of Morphology, Department of Histology and Embryology, School of Medicine, University of Mostar, 88 000 Mostar, Bosnia and Herzegovina; deni.ramljak@mef.sum.ba (D.R.); martina.vukoja@mef.sum.ba (M.V.); maja.pivic@mef.sum.ba (M.B.); una.glamoclija@mef.sum.ba (U.G.); 2 Health Care Center Mostar, 88 000 Mostar, Bosnia and Herzegovina; 3 Faculty of Health Studies, University of Mostar, 88 000 Mostar, Bosnia and Herzegovina; marina.curlin@fzs.sum.ba (M.C.); olivera.peric@yahoo.com (O.P.); 4 Department of Anatomy, Histology and Embryology, School of Medicine, University of Split, Šoltanska 2, 21000 Split, Croatia; 5 Faculty of Pharmacy, University of Sarajevo, 71 000 Sarajevo, Bosnia and Herzegovina; 6 Health Care Center Stari Grad Mostar, 88 000 Mostar, Bosnia and Herzegovina; bejana.purisevic@mef.sum.ba

**Keywords:** COVID-19, CD8, effector memory, perforin, granzymes, granulysin, IFN-γ, FASL

## Abstract

Healthy and controlled immune response in COVID-19 is crucial for mild forms of the disease. Although CD8+ T cells play important role in this response, there is still a lack of studies showing the gene expression profiles in those cells at the beginning of the disease as potential predictors of more severe forms after the first week. We investigated a proportion of different subpopulations of CD8+ T cells and their gene expression patterns for cytotoxic proteins (perforin-1 (PRF1), granulysin (GNLY), granzyme B (GZMB), granzyme A (GZMA), granzyme K (GZMK)), cytokine interferon-γ (IFN-γ), and apoptotic protein Fas ligand (FASL) in CD8+ T cells from peripheral blood in first weeks of SARS-CoV-2 infection. Sixteen COVID-19 patients and nine healthy controls were included. The absolute counts of total lymphocytes (*p* = 0.007), CD3+ (*p* = 0.05), and CD8+ T cells (*p* = 0.01) in COVID-19 patients were significantly decreased compared to healthy controls. In COVID-19 patients in CD8+ T cell compartment, we observed lower frequency effector memory 1 (EM1) (*p* = 0.06) and effector memory 4 (EM4) (*p* < 0.001) CD8+ T cells. Higher mRNA expression of PRF1 (*p* = 0.05) and lower mRNA expression of FASL (*p* = 0.05) at the fifth day of the disease were found in COVID-19 patients compared to healthy controls. mRNA expression of PRF1 (*p* < 0.001) and IFN-γ (*p* < 0.001) was significantly downregulated in the first week of disease in COVID-19 patients who progressed to moderate and severe forms after the first week, compared to patients with mild symptoms during the entire disease course. GZMK (*p* < 0.01) and FASL (*p* < 0.01) mRNA expression was downregulated in all COVID-19 patients compared to healthy controls. Our results can lead to a better understanding of the inappropriate immune response of CD8+ T cells in SARS-CoV2 with the faster progression of the disease.

## 1. Introduction

A new disease named Coronavirus Disease 2019 (COVID-19) was diagnosed for the first time in December 2019 in patients with acute respiratory syndrome in Wuhan. Since then, it spread rapidly and became a world-wide problem [1]. By the end of October 2021, over 243 million COVID-19 cases and over 4.9 million COVID-19 deaths were confirmed [2].

Studies in Wuhan show that around 60% of COVID-19 patients suffered from lymphopenia, and patients who died of COVID-19 had a lower number of lymphocytes than patients who survived [3]. Other studies also showed that lymphopenia is associated with the severity of the disease [4,5]. Specifically, CD8+ T cell, but not CD4+ T cell, reduction by SARS-CoV-2 is associated with a worse prognosis in COVID-19 patients [6]. Higher CD8+/CD4+ T cell ratios can be found in mild disease, whereas severe disease displayed the reverse trend [7,8]. However, a significant difference between the critical cases and healthy controls in terms of CD8+ T cell counts has been observed in recent literature, but there was no significant difference between the critical and mild cases, while the percentage of CD8+ T cell counts was significantly lower in mild patients compared to controls [9,10].

Similarly, AIDS patients also have a reduced number of peripheral blood lymphocytes (PBL). While HIV (human immunodeficiency virus) can directly kill T cells and change the CD4+/CD8+ ratio, SARS-CoV-2 cannot [11].

There are four major subpopulations of T cells: naïve, central memory (CM), effector memory (EM), and effector (E) [12,13]. In COVID-19 patients, more effector phenotype was found in CD8+ T cells, while CD4+ T cells had more central phenotype [14]. EM and effector CD8+ T cells are mainly cytotoxic, and they express granzyme A (GZMA), granzyme B (GZMB), perforin (PRF1), and granulysin (GNLY). GZMB is an enzyme that splits and activates caspase enzymes, while PRF1 is required for delivery of granzyme to the cytoplasm of the target cell [15]. Besides cytotoxic activity, GNLY serves as a distinguishing biomarker of cell-mediated immunity, infection, and graft versus host disease (GVHD) [11]. GZMA and GZMK are cytotoxic proteins appearing as an alternative cytotoxic path and are mainly expressed in effector memory T cells [16,17]. Effector cytotoxic T cells also produce interferon-gamma (IFN-γ), which is included in numerous immunological functions and inflammatory processes [18]. Necrosis Fas and Fas Ligand (FASL) belong to the tumor factor family, and their interaction initiates apoptosis in virus infected cells [19]. Immune cells during infection remain active by increased expression of PRF1 and granzymes, despite the reduction in the total number of cells [18]. It is still not known how effector cytotoxic T cells mediate viral containment. In HIV patients, perforin-mediated cytotoxicity and polyfunctionality is defined by simultaneous production of chemokines and cytokines, which correlates to immune-mediated protection from disease progression [19,20,21].

In clinical practice, it has been observed that mild COVID-19 patients had a stable disease; however, some of the patients suddenly became severe in the course of seven to ten days with the worsening of respiratory symptoms. Lymphocyte count in COVID-19 patients differed during the weeks of infection [22].

The aim of our study was to investigate the gene expression pattern of cytotoxic proteins (PRF1, GZMB, GNLY, GZMA, GZMK), cytokine (IFN-γ), and apoptotic protein (FASL) in CD8+ T cells from peripheral blood, and the proportion of different subpopulations of CD8+ T cells in first weeks of SARS-CoV-2 infection. The aim was to provide a comprehensive view of the effector molecules produced by CD8+ T cells from peripheral blood and elucidate the importance of their shift in the early viral pattern of COVID-19.

## 2. Materials and Methods

### 2.1. Study Population, Setting, and Data Collection

This prospective controlled study was approved by the Ethical Committee of the Health Care Centre Mostar in accordance with the Declaration of Helsinki and Good Clinical Practice. Informed consent was obtained from all patients and healthy controls. The patients participating in the study were admitted to the Health Care Centre Mostar in Mostar, Bosnia and Herzegovina in the period from February to June 2021.

The inclusion criteria for COVID-19 patients were mild symptoms from the first day of the disease, according to guidelines from the National Institutes of Health [23] and confirmed COVID-19 by PCR testing. Prior to PCR confirmed diagnosis, patients were given symptomatic therapy. The healthy control were subjects without COVID-19 (negative PCR and serological test), unvaccinated, and without any other inflammatory or immunological disease. The non-inclusion criteria were immunological disorders, inflammatory disease other than COVID-19, severe and critical COVID-19 at the time of diagnosis, SARS-CoV-2 vaccination, previous COVID-19, and pregnancy. The first day when symptoms appeared was considered the first day of the disease. The transition from mild to moderate or severe COVID-19 was evaluated (Supplementary Figure S1).

### 2.2. Real Time PCR

Real Time PCR analysis was performed on an Applied Biosystems Fast 7500 Real-Time PCR instrument. Blood samples containing 5 mL of blood were obtained from all patients in an ethylenediamine tetraacetic acid (EDTA) tube. Mononuclear cells were isolated from peripheral blood using the Lymphoprep^TM^ protocol (STEMCELL Technologies, Vancouver, BC, Canada), according to the manufacturer’s instructions. After isolation, mononuclear cells were counted, and the total number had to be higher than 1 × 10^8^/mL in the volume of 0.1 to 2.5 mL. An EasySep^TM^ Human CD8 Positive Selection Kit II (18053, STEMCELL Technologies, Vancouver, BC, Canada) was used for isolation of CD8+ T cells. The purity of the selected CD8+ T cells was confirmed by flow cytometry. The resulting CD8+ T cells were >98%, as determined by immunofluorescence analysis with directly labeled mAb. Isolated CD8+ T cells were used for total RNA extraction by QIAamp RNA Blood Mini kit (QI52304, Qiagen, Hilden, Germany), according to the manufacturer’s instruction. RNA concentrations were determined by Qubit (Thermo Scientific, Waltham, MA, USA). Total RNA extracted from CD8+ T cells was used for qPCR analysis. The prepared RNA was reverse transcripted into cDNA using High-Capacity cDNA Reverse Transcription Kits (Applied Biosystems, Waltham, MA, USA).

With the cDNA (2.16 ng) as the template, PCR was performed using a QuantiNova PCR Kit (Qiagen, Germany). For real-time PCR, PCR mixes contained 5.1 µL H2O, 1.4 µL forward primers, 1.4 µL reverse primers, 10 µL Syber green mix, and 0.1 µL QN ROX Reference Dye. A negative control using nuclease-free water instead of the cDNA template was included in each experiment. Sequences for forward and reverse primers are presented in Supplemental Table S1. Analysis of gene expression changes was performed by the comparative 2^−ΔΔCT^ method. Results are expressed as the relative gene expression (relative to healthy control), as a fold change. Data were normalized to a housekeeping reference gene (GAPDH). mRNA expression of GAPDH was normally distributed with lower standard deviation compared to mRNA expression of beta actin.

### 2.3. Lymphocyte Subpopulation Test

Fasting whole blood from every patient was collected in EDTA collection tubes. Whole blood was incubated with BD Multitest 6-color TBNK reagent and then lysed with BD FACS™ lysing solution. Lymphocyte subpopulations were acquired and analyzed with BD FACSCanto Clinical Software. The BD Multitest 6-color TBNK reagent contains the following antibodies to identify and count different lymphocyte subsets: CD3 FITC, CD16 PE, CD56 PE, CD45 PerCP-Cy™5.5, CD4 PE-Cy™7, CD19 APC, and CD8 APC-Cy7. The results are presented as absolute count (cells/µL).

### 2.4. Immunophenotyping

An amount of 100 µL of whole blood was stained for surface markers CCR7-FITC, CD28-PE, CD45RA-PE-Cy™7, CD27-PerCP-Cy™5.5, CD8-APC, and CD3-APC-H7 (obtained from BD Biosciences, USA). Cells were acquired on a CANTO II flow cytometer (BD Biosciences, San Jose, CA, USA), where at least 1,000,000 events in the lymphocyte scatter gate were acquired. Staining specificity was confirmed using fluorescence minus one (FMO), all antibodies minus one. Data analysis was carried out using DIVA software.

### 2.5. Statistical Analysis

Statistical analysis was performed using GraphPad software (San Diego, CA, USA). Results were considered statistically significant at the confidence level α = 0.05, i.e., when *p* < 0.05. Normality of data distribution was analyzed with D’Agostino–Pearson, Shapiro–Wilk, and Kolmogorov–Smirnov tests. Statistical significance was evaluated with unpaired *t*-test, one-way ANOVA, and Tukey post-hoc test or the Kruskal–Wallis and Dunn’s post-hoc tests. Data is presented as value and percentage from the total number, mean value ± standard deviation for variables with normal distribution, and median and interquartile range for ordinal variables and variables with non-normal distribution.

## 3. Results

### 3.1. Patients’ Baseline Characteristics

Nine healthy controls and 16 SARS-CoV-2 positive patients were included in the study. The clinical and demographic data are presented in Table 1.

### 3.2. Gene Expression in CD8+ T Cells from PBL of Mild COVID-19 Patients in the First Week of Illness Compared to Healthy Controls

**Figure 1:**
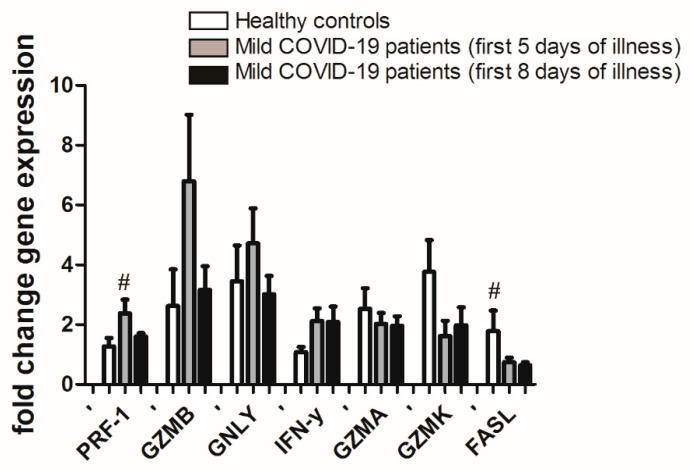
qPCR mRNA fold change of PRF-1, GZMB, GNLY, IFN-γ, GZMA, GZMK, and FASL in CD8+ T cells from peripheral blood of mild COVID-19 patients at the fifth and eight day of the disease (*n* = 12) compared to heathy controls (*n* =9). Data is presented as mean value ± standard deviation. ^#^ *p* value 0.05 (Two-way ANOVA followed by Tukey’s multiple comparison test).

Real-time PCR for PRF1, GZMB, GNLY, IFN-γ, GZMA, GZMK, and FASL mRNA was performed from total RNA isolated from CD8+ T cells. mRNA for all of the cytotoxic proteins mentioned above was identified in all samples. There was no statistically significant difference in mRNA expression for all cytotoxic proteins in patients with COVID-19 between the fifth and eighth day of the disease. However, a trend of higher mRNA expression of cytotoxic protein PRF1 and GZMB in COVID-19 patients at the fifth day of the disease was noticed (Figure 1). In contrast, FASL and GZMK showed a trend of decreased mRNA expression at the fifth day of the disease in COVID-19 patients compared to the healthy control (Figure 1).

### 3.3. Lymphopenia with Relative Decreases of CD8+ Effector Memory Subsets Define First Week of COVID-19 Infection

**Figure 2:**
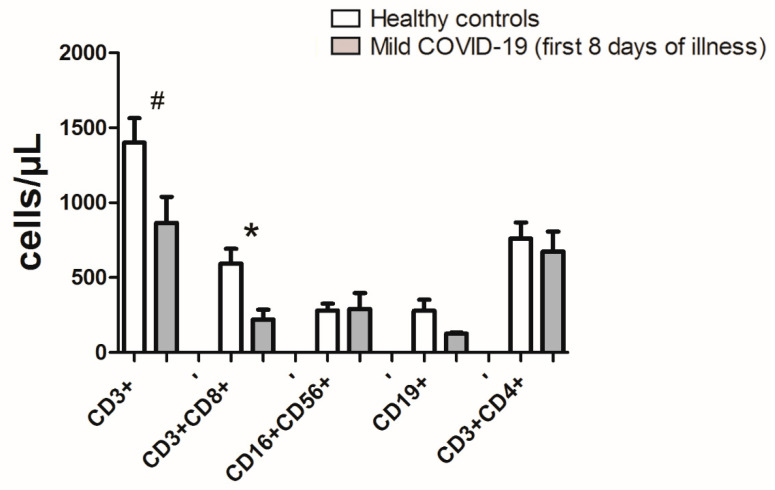
Absolute count of CD3+, CD3+CD8+, CD16+CD56+, CD19+, and CD3+CD4+ in healthy controls and mild COVID-19 patients within first eight days of illness. Data is presented as mean value ± standard deviation. Significant differences are indicated by *p* * = 0.01, *p* ^#^ = 0.05 (unpaired *t* test with Welch’s correction).

The absolute counts of CD3+ (mean = 866; SD ± 345.5) and CD8+ T cells (mean= 221.1; SD ± 130.4) in COVID-19 patients were significantly decreased compared to heathy controls (CD3 + mean 1401; SD ± 43.6), *p* = 0.05, (CD8+ mean 594.8; SD ± 260.1), *p* = 0.01. Additionally, no statistically significant differences were found for B cells (mean 125.1; SD ± 15.8), NK cells (mean 290.3; SD ± 187), or CD4 + (mean 674.3; SD ± 230.3) compared to heathy control B cells (mean 278.7; SD ± 182.6), NK cells (mean 281.2; SD ± 110.7), or CD4 (mean 760.9; SD ± 263.8) (Figure 2).

**Figure 3:**
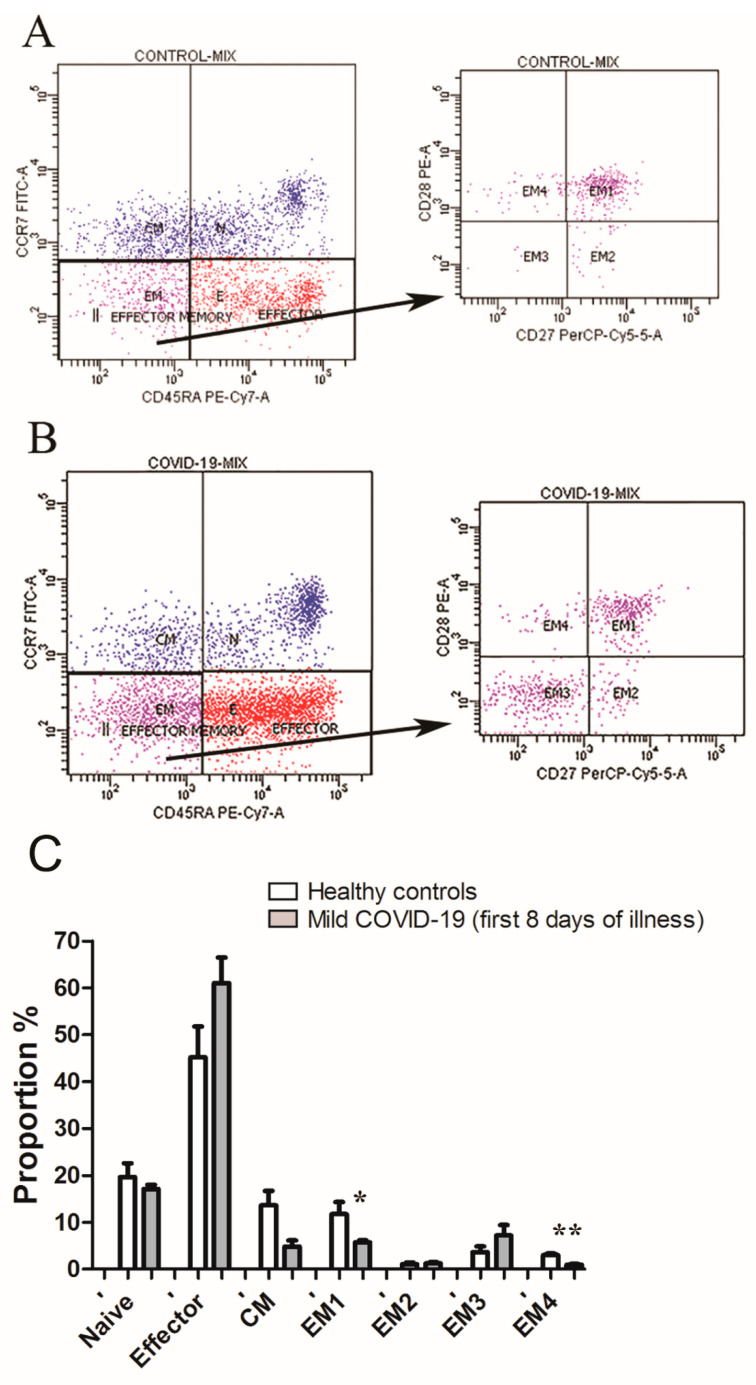
Flow cytometry analysis. (**A**) Differential expression of CD45RA, CCR7, CD27, and CD28 cell surface markers on total CD8+ T cells from healthy control (*n* = 9) and (**B**) COVID-19 patients (*n* = 12). CD3+CD8+ gated cells were separated into four subsets (naive, CM, EM, and effector) based on CD45RA and CCR7 labeling. Effector memory (purple) were analyzed for CD27 and CD28 co-expression, and the proportion of EM1, EM2, EM3, and EM4 cells was determined. (**C**) Proportion of naïve, effector, CM, EM1, EM2, EM3, and EM4 CD8+ T cells in healthy controls and mild COVID-19 patients within first eight days of illness. Data is presented as mean value ± standard deviation. Significant differences were indicated by *p* * = 0.06 and *p* ** < 0.001 (unpaired t test with Welch’s correction).

Based on flow cytometry, CD8+ T cells were classified into four functionally different populations: naïve (RA+CCR7+), effector (RA+CCR7−), CM (RA−CCR7+), and EM (RA−CCR7+). Further analysis revealed four subsets of EM cells: EM1 (28+27+), EM2 (28−27+), EM3 (28−27−), and EM4 (28+27−). Flow cytometry analysis revealed that the majority of CD8+ T cells from COVID-19 patients and healthy controls were naïve and effector (Figure 3).

In COVID-19 patients, in the CD8+ T cell compartment, a lower frequency of EM1 and EM4 CD8+ T cells were observed compared to healthy controls (Figure 3).

### 3.4. Gene Expression in CD8+ T Cells from PBL of COVID-19 Patients in the First Eight Days of Disease, Depending on the Severity of Disease after Ten Days (Mild versus Moderate and Severe) Compared to Healthy Controls

Among 16 recruited COVID-19 patients, at ten days after the diagnosis, seven patients developed moderate or severe clinical appearance (four were moderate, three were severe) (Table 2).

**Figure 4:**
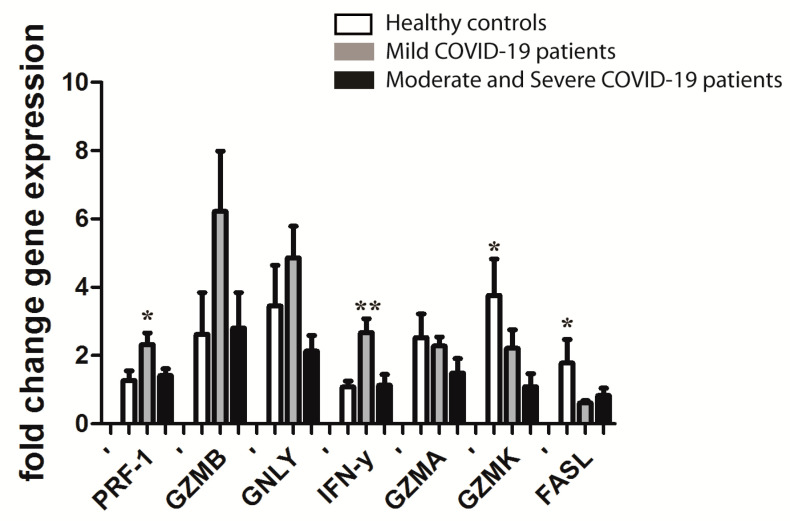
Real-time PCR mRNA fold change of PRF1, GZMB, GNLY, IFN-γ, GZMA, GZMK, and FASL in CD8+ T cells from the peripheral blood of three groups of analyzed participants (stabile mild COVID-19 patients, *n* = 9; COVID-19 patients progressed to moderate and severe disease after 10th day, *n* = 7; and healthy controls, *n* = 9). Data is presented as mean value ± standard deviation. Significant differences are indicated by *p* * <  0.01, *p* **  <  0.001 (one-way ANOVA test followed by Tukey or Kruskal–Wallis test followed by Dunn’s multiple comparison test).

mRNA expression of PRF1 and IFN-γ was significantly downregulated in the first week of the disease in COVID-19 patients who progressed after the tenth day of illness to moderate and severe compared to patients with mild symptoms after the tenth day of illness. GZMK and FASL mRNA expression was downregulated in all COVID-19 patients compared to healthy controls. Furthermore, there was a trend toward higher mRNA expression of other cytotoxic proteins, GNLY and GZMB, in CD8+ T cells in mild COVID-19 patients with stable clinical appearance (Figure 4).

## 4. Discussion

In this study, we analyzed the gene expression pattern of cytotoxic proteins (PRF1, GZMB, GNLY, GZMA, GZMK), cytokine (IFN-γ), and apoptotic protein (FASL) in CD8+ T cells from the peripheral blood of COVID-19 patients. The results revealed differences between the fifth day of the disease compared to control and between patients with stabile mild disease and patients who had deterioration after the tenth day of the disease compared to control. Additionally, changes in the proportion of different subpopulations of CD8+ T cells between COVID-19 patients within the first eight days of illness and controls were observed. Therefore, some genes show a different trend at the fifth and eighth day of COVID-19. PRF1, GZMB, and GNLY genes expressed at the fifth day of infection were upregulated. On the eight day, gene expression was similar to controls, and this finding can be used for a prediction of COVID-19 progression.

The viral load of SARS-CoV-2 is highest during the first three to five days of illness, and it declines thereafter [24]. Therefore, in this study, we examined the first week of COVID-19 in time peaks of the fifth and eighth day of the disease. The time gap between the peak of viral load and the worsening of clinical appearance implies the importance of immune response in the pathogenesis of severe COVID-19. The impaired immunopathogenesis of severe COVID-19 should be recognized in a timely manner. Multiple lines of evidence indicate that the unbalanced pro-inflammatory response is implicated in the progression of the disease [25]. Similarly to some previous studies, we observed increased peripheral blood levels of immune-inflammatory parameters such as AST, LDH, CRP, IL6, and PT in COVID-19 patients compared to controls [26,27,28].

Within the first week of the disease, we found significantly increased gene expression of cytotoxic protein PRF1 in COVID-19 patients compared to healthy controls. Cytotoxic proteins (GZMB and GNLY) and cytokine IFN-γ showed a trend toward higher expression without statistical significance. Contrary to this, proapoptotic FASL was significantly decreased, while GZMK showed a trend towards lower expression without statistical significance. These findings were in line with flow cytometry results where an increased proportion of effector CD8+ T cells and a decreased proportion of effector memory CD8+ T cells that produce GZMK was observed. This indicated distinct downregulation of CD8+ T cells in the first week of the disease. Furthermore, differences in cytotoxic protein expression might imply differential regulation at the transcriptional and post-transcriptional level in COVID-19 patients. When we correlated our findings with the outcome of COVID-19, there was a significant decrease in the expression of PRF1 and IFN-γ in CD8+ T cells in patients who developed moderate and severe COVID-19 compared to those who remained in the mild group after the tenth day of illness. GZMB expression was also decreased in patients who deteriorated after the tenth day of the disease when compared to the mild group, but with no statistical significance. FASL and GZMK expression remained low in comparison to healthy controls, regardless of the severity of the disease.

Several studies found similar results. Ahmadi et al., in a study on 14 COVID-19 patients using intracellular cytokine straining, found increased expression of GZMB and PRF1 in CD8+ T cells from COVID-19 patients. They also reported a reduced number of CD4+ T cells, CD8+ T cells, and B cells in COVID-19 patients compared to healthy controls [29]. Mazzoni et al. reported no difference in expression of GZMA in CD8+ T cells between COVID-19 patients and healthy controls. Furthermore, they found increased expression of PRF1, but without statistical significance [30]. Other studies also reported increased PRF1, GNLY, soluble Fas, and GZMB in COVID-19 patients [30,31]. Similar to our results, Li et al. reported that expression of PRF1 and GZMA in mild cases was increased compared to severe cases and healthy controls, but they found no difference in expression for soluble FASL, IFN-γ, GNLY, soluble FAS, or GZMB [32]. Contrary to our results, Kang et al. report that expression of PRF1 and GZMB in CD8+ T cells was lower in the mild group [33]. Other studies report that expression of PRF1 and GZMB in CD8+ T cells increases with the progression of the disease [34]. The reason for this might be caused by a different time point of peripheral blood sample collection and a different stage of the disease. Our results can lead to better understanding of the inappropriate immune response of CD8+ T cells in SARS-CoV2 with the deterioration of the disease.

Serum IFN-γ levels are higher in patients with COVID-19 compared with healthy individuals [35]. Higher IFN-γ levels are associated with greater viral load and damage in the lungs [36].

The nature of CD8+ T cells reactive to coronaviridae is quite different from those that respond to the influenza virus, RSV (respiratory syncytial virus), or HIV [37]. CD8+ T cells differentiate from naive into terminally differentiated effector or EM T cell subpopulations in response to viral antigen exposure. In the acute phase of antiviral immune response, there is a vast majority of effector and EM cell populations. Some of these subpopulations might already be functionally exhausted during acute COVID-19.

The EM CD8+ T cells are key gatekeepers of tissues that are susceptible to pathogenic microorganisms’ invasion. On the other hand, for accelerated immune reaction, CD8+ CM T cells are recruited into the secondary lymphoid organs [38]. The proportion of EM (CCR7+, CD45RA−, CD28+ and CD27+/−) CD8+ T cells known to have higher concentration of GZMK is significantly decreased in the first week of COVID-19. Kratzer et al. found that, in COVID-19 convalescent patients, CD3+CD8+ EM cells were increased in comparison to healthy controls [39]. This might imply aberrant cytotoxic response in the first week of the disease.

For the first time, in this study we showed that FASL is decreased in CD8+ T cells in the first week of COVID-19. Only one study, Kusnadi et al. using the NGS method, showed similar results with decreased transcripts encoding for cytotoxicity molecules, including FASL in mild COVID-19 patients. This might imply the possibility that the exhaustion of CD8+ T cells is clinically important for the limitation of excessive tissue damage caused by SARS-CoV-2 CD8+ T cells [40]. Tavukcuoglu et al. found that patients recovered from COVID-19 had FASL secretion enhanced in CD4^+^ and CD8^+^ effector memory and central memory T cells. This finding was correlated with functional capacities to strengths anti-viral immunity against SARS-CoV-2 [38]. In our study, an early decrease in the expression of FASL in CD8+ T cells might contribute to viral pathogenesis in COVID-19 disease.

In conclusion, contrary to nonspecific immunity and virus neutralization by antibodies, only T cells can recognize virus fragments after the host cell is infected. In our study, we found a decreased number of EM 1 and 4 CD8+ T cells and a decreased level of GZMK and FASL in all COVID-19 patients, regardless of the disease progression. PRF1 and IFN-γ mediated response was appropriate only in patients without disease progression. The question remains of whether and how altered expression of these genes leads to CD8+ T cell dysregulation. We suggest that the main features of severe COVID-19 disease are a consequence of reduced innate antiviral defense due to attenuated responses of IFN-γ in CD8+ T cells.

## Figures and Tables

**Table 1:** Baseline characteristics of COVID-19 patients and healthy controls.

	COVID-19 *n* = 16	Healthy Control *n* = 9	*p* Value
Age (years) (mean ± SD)	51 ± 13	45 ± 10	0.678
Gender (M/F) (*n*, %)	7 (44%)/9 (56%)	4 (44%)/5 (56%)	0.563
Comorbidities (*n*, %)	7 (44%)	2 (22%)	0.040
Thrombocytes(×10^9^/L) (mean ± SD)	191 ± 71	255 ± 62	0.062
Leukocytes (×10^9^/L) (mean ± SD)	4.3 (3.8−4.9)	5.9 ± 1.7	0.076
Neutrophils (×10^9^/L) (mean ± SD)	2.4 (1.6−3.3)	3.32 ± 1.16	0.285
Lymphocytes (×10^9^/L) (mean± SD)	1.3 ± 0.5	1.9 ± 0.5	0.007
Neutrophils/Lymphocytes (median; IQR)	1.7 (1.1−3.1)	1.7 (1.5–1.9)	0.664
Leukocytes/Lymphocytes (mean ± SD)	3.2 (2.6−4.8)	3.1 ± 0.6	0.462
Urea (mmol/L) (median; IQR)	4.2 (3.7−4.6)	5.0 (4.8–6.0)	0.015
AST (U/L) (mean ± SD)	29 (26−39)	20 ± 7	0.016
ALT (U/L) (mean ± SD)	25 (19−46)	22 ± 10	0.151
LDH (U/L) (median; IQR)	226 ± 73	151 (144–168)	0.005
CRP (mg/L) (median; IQR)	9.9 (3.7−45.2)	1.1 (0.6–1.3)	<0.001
IL6 (pg/mL) (median; IQR)	5.9 (2.1−35.9)	1.5 (1.5–1.5)	<0.001
Ferritin (µg/L) (mean ± SD)	129.0 (86.0−245.0)	122.5 ± 176.6	0.204
d-dimer (mg/L) (mean ± SD)	0.52 ± 0.27	0.47 ± 0.16	0.626
PT (ng/mL) (median; IQR)	0.04 (0.02−0.06)	0.01 (0.01–0.02)	0.003

Abbreviations: AST: Aspartate transaminase; ALT: Alanine aminotransferase; CRP: C-reactive protein; IL6: interleukin 6; IQR: interquartile range; LDH: Lactate dehydrogenase; M: mild; MS: moderate and severe; SD: standard deviation; PT: prothrombin time.

**Table 2:** Baseline characteristics of control, patients who had stable mild COVID-19, and stable moderate and severe COVID-19.

					Post Hoc Tests *p* Value		
	Control (*n* = 9)	Mild COVID-19 (*n* = 9)	MS COVID-19 (*n* = 7)	*p* Value	Mild vs. MS	Mild vs. Control	MS vs. Control
Age (years) (mean ± SD)	45 ± 10	46 ± 12	58 ± 12	0.073			
Gender (M/F) (*n*, %)	5 (56%)/4 (44%)	3 (33%)/6 (67%)	4(57%)/3 (43%)	0.558			
Comorbidities (*n*, %)	2 (22%)	3 (33%)	4 (57%)	0.628			
Thrombocytes (×10^9^/L) (mean ± SD)	255 ± 62	209 ± 56	167 ± 84	0.063			
Leukocytes (×10^9^/L) (mean ± SD)	5.9 ± 1.7	4.1 ± 0.9	5.3 ± 2.5	0.108			
Neutrophils (×10^9^/L) (mean ± SD)	3.32 ± 1.16	2.12 ± 0.98	3.56 ± 2.21	0.133			
Lymphocytes (×10^9^/L) (mean ± SD)	1.9 ± 0.5	1.4 ± 0.4	1.2 ± 0.5	0.024	0.765	0.087	0.049
Neutrophils/Lymphocytes (median; IQR)	1.7 (1.5–1.9)	1.8 (1.4–2.5)	3.1 (2.4–3.9)	0.231			
Leukocytes/Lymphocytes (mean ± SD)	3.1 ± 0.6	3.2 ± 1.4	4.5 ± 1.8	0.127			
Urea (mmol/L) (median; IQR)	5.0 (4.8–6.0)	4.2 (4.0–4.4)	3.7 (3.3–5.7)	0.066			
AST (U/L) (mean ± SD)	20 ± 7	26 ± 7	44 ± 17	<0.001	0.061	0.183	0.018
ALT (U/L) (mean ± SD)	22 ± 10	27 ± 13	42 ± 27	0.083			
LDH (U/L) (median; IQR)	151 (144–168)	156 (145–207)	299 (284–330)	0.001	0.017	1.000	0.001
CRP (mg/L) (median; IQR)	1.1 (0.6−1.3)	3.8 (3.1−6.0)	53.7 (16.8–109.0)	<0.001	0.126	0.049	<0.001
Ferritin (µg/L) (mean ± SD)	122.5 ± 176.6	122.4 ± 89.3	361.7 ± 337.4	0.067			
d-dimer (mg/L) (mean ± SD)	0.47 ± 0.16	0.38 ± 0.22	0.71 ± 0.21	0.011	0.021	0.604	0.076
PT (ng/mL) (median; IQR)	0.01 (0.01–0.02	0.03 (0.02–0.04)	0.08 (0.04−0.12)	0.003	0.315	0.137	0.002
IL6 (pg/mL) (median; IQR)	1.5 (1.5−1.5)	2.1 (1.7−4.5)	50.2 (23.4−56.1)	<0.001	0.046	0.141	<0.001

Abbreviations: AST: Aspartate transaminase; ALT: Alanine aminotransferase; CRP: C-reactive protein; IL6: interleukin 6; LDH: Lactate dehydrogenase; MS: moderate and severe; SD: standard deviation; PT: prothrombin time.

## Data Availability

The datasets used and/or analyzed during the current study are available from the corresponding author on request.

## Supplementary Materials

The following are available online at https://www.mdpi.com/article/10.3390/jpm11121291/s1, Table S1. Primer sequences of selected target genes. Figure S1. Schematic overview of study design.
