# Supplementary material for: Early Response of CD8+ T Cells in COVID-19 Patients
Source: J Pers Med. 2021 Dec 3;11(12):1291. doi: 10.3390/jpm11121291 (PMC8704882; doi:10.3390/jpm11121291)
Supplement: Supplementary file 1 [file jpm-11-01291-s001.zip › jpm-1468358-Supplementary.pdf]

Table S1.Table shows forward and reverse primer sequences of transcripts used in Real-Time PCR.

Transcript	Forward primer	Reverse primer
**Beta actin**	ACCACACCTTCTACAATGAG	TAGCACAGCCTGGATAGC
**GAPDH**	ACCCACTCCTCCACCTTTGAC	TCCACCACCCTGTTGCTGTAG
**Perforin**	GGTTCACTGCCACGGATG	ACAGGTGCCAAGGAGGTC
**Granulysin**	GAAGAAGATGGTGGATAAG	CTAGACTGATACCTCCTC
**Granzyme A**	ATGGTCCTACTTAGTCTTG	CCTGGTTATTGAGTGAGC
**Granzyme B**	CTGATACGAGACGACTTC	GGATTATAGGCTGGATGG
**Granzyme K**	TTAAGACCTTCTGACACC	TGGAAGACACCTTTACAG
**IFN-γ**	AGCTCTGCATCGTTTTGGGTT	GTTCCATTATCCGCTACATCTGAA
**FASL**	TGGCCTTGTGATCAATGAAA	TCATCATCTTCCCCTCCATC

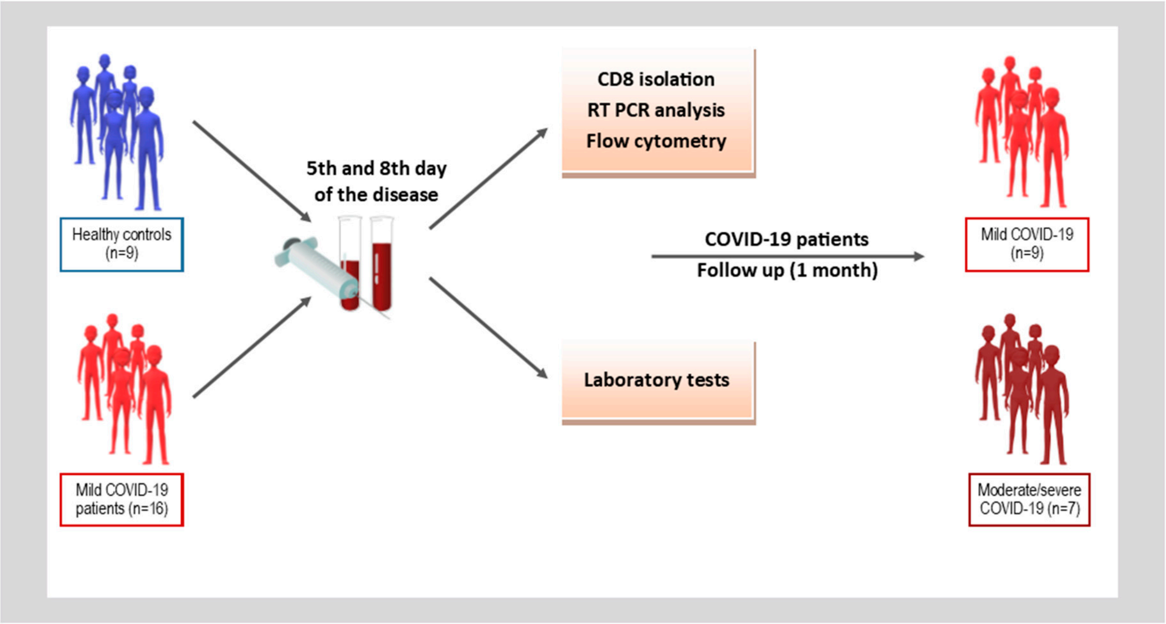

Supplemental Figure S1. Schematic overview of study design. Longitudinal blood samples were taken from patients on the 5th and 8th day after first symptoms of SARS-CoV-2 infection.
